# Development of pH-responsive gelatin/PVP nanogel by gamma radiation for controlled delivery of silibinin

**DOI:** 10.1038/s41598-026-58355-8

**Published:** 2026-06-26

**Authors:** Zakaria Mohamed Alghzzawy, Mohammed Hussein Awwad, Tarek Khaled Elmaghraby, Sanaa Abd El-Hamid Hagag, Azza Elsayed Kayed, Mohamed Mohamady Ghobashy, Ahmed M. Elbarbary, Doaa Sabry Ibrahim

**Affiliations:** 1 Radiation Biology Research Department, National Center for Radiation Research and Technology, Egyptian Atomic Energy Authority, Cairo, Egypt; 2 Zoology Department, Faculty of Science, Benha University, Benha, Egypt; 3 Polymer Chemistry Department, National Center for Radiation Research and Technology, Egyptian Atomic Energy Authority, Cairo, Egypt

**Keywords:** Silibinin, Gelatin, Polyvinylpyrrolidone, Nanogel, Gamma-radiation, Controlled release, HepG2 cells, Biochemistry, Biotechnology, Chemistry, Drug discovery, Materials science

## Abstract

Silibinin (SB) is a natural polyphenolic flavonoid with recognized health and therapeutic potential. SB has poor aqueous solubility, which limits its bioavailability and therapeutic effectiveness. To overcome these limitations, a pH-responsive gelatin/polyvinylpyrrolidone (Gel/PVP) nanogel was synthesized via γ-irradiation (5 kGy) for controlled SB delivery. SB/PVP solid dispersions were first prepared by solvent evaporation to enhance dissolution, then incorporated into the preformed Gel/PVP nanogel in the presence of N-hydroxysuccinimide (NHS), yielding the SB/Gel/PVP formulation. FTIR confirmed effective crosslinking between gelatin and PVP and successful SB encapsulation, while XRD revealed an amorphous state favorable for solubility. TEM micrograph of SB/Gel/PVP showed cubic nanoparticles < 50 nm, with a zero-point charge at pH 5.5. The nanogel achieved SB entrapment efficiency of 82% and a loading capacity of 3.34%. In vitro release studies demonstrated pH-responsive behavior, with SB release threefold higher than free drug after 6 h, and the highest release was observed at pH 4.5. The MTT assay confirmed that the SB/Gel/PVP nanogel significantly inhibited HepG2 cell proliferation compared with free SB (*P* < 0.05). Moreover, the biochemical and histopathological analyses of liver and kidney tissues in rats treated with SB or SB/Gel/PVP (25 mg/kg) indicated good biocompatibility. Collectively, these findings highlight Gel/PVP nanogels as a promising platform for sustained and targeted SB delivery in pharmaceutical applications.

**Supplementary Information:**

The online version contains supplementary material available at 10.1038/s41598-026-58355-8.

## Introduction

The development of advanced drug delivery systems has become an important area of research for improving the therapeutic efficacy and bioavailability of poorly water-soluble drugs. pH-sensitive materials are smart systems that undergo structural or physicochemical changes in response to environmental acidity or alkalinity. They can swell, collapse, or reorganize their networks, and respond to diverse stimuli, including pH and temperature, making them highly valuable in biomedical applications such as drug delivery and wound dressings^1^. pH-responsive hydrogels have been successfully applied in wound healing, antimicrobial therapy, and tumor treatment ^2,3^. Among these systems, nanogels are nanoscale hydrogel particles formed by crosslinked polymer networks that can swell in aqueous environments while maintaining structural integrity. Stimuli-responsive nanogels have emerged as promising carriers for site-specific drug delivery. These systems can alter their swelling behavior and drug release profile in response to changes in environmental pH, enabling selective release in acidic or pathological tissues such as tumor sites and inflamed regions. The pH-sensitive nanogels can therefore enhance therapeutic efficiency while reducing adverse side effects associated with conventional drug administration. Their ability to enhance solubility, dissolution, and controlled release of poorly water-soluble drugs underscores their potential as versatile platforms for modern nanomedicine^4^.

Various methods, such as emulsion polymerization, chemical crosslinking, and radiation-induced polymerization, have been employed for nanogel synthesis. Gamma irradiation enables the formation of pH-sensitive hydrogels and nanogels with enhanced stability, reproducibility, and biomedical utility^5–7^, because it offers a clean, efficient, and controllable crosslinking method compared to traditional chemical crosslinking methods ^8^. Moreover, various polymeric materials have been synthesized for diverse applications using this approach, which provides controlled crosslinking without chemical initiators and ensures simultaneous sterilization^5,9–15^. Moreover, irradiation allows precise control over crosslink density, enabling fine-tuning of swelling behavior and pH responsiveness. Natural and synthetic polymer combinations are widely employed in nanogel fabrication to achieve optimal mechanical stability, drug encapsulation efficiency, and controlled release behavior. Gelatin, a natural biopolymer derived from collagen, plays a pivotal role in nanogel design due to its biocompatibility, biodegradability, and amphoteric nature. The presence of ionizable functional groups, including carboxyl (–COOH), amino (–NH₂), and hydroxyl (–OH), enables pH-responsive behavior, dynamic conformational changes, chemical modifications, and crosslinking, improving drug encapsulation and controlled release. These properties make gelatin an ideal candidate for nanogels responsive to stimuli, capable of reversible swelling or shrinking in response to environmental triggers such as pH, ionic strength, and temperature. The coexisting acidic and basic residues enables dynamic modulation of the net charge of the polymer based on the surrounding medium. This charge variation, which depends on the pH, directly governs nanogel dimensions and swelling behavior, facilitating targeted and controlled drug release. Furthermore, the abundant functional groups of gelatin mediate hydrogen bonding, electrostatic interactions, and covalent coupling with therapeutic agents^16,17^. Collectively, these mechanisms maximize encapsulation efficiency, shield payloads from degradation, and enhance drug solubility and bioavailability. These superior properties have significantly broadened the utility of gelatin across diverse fields, including bioengineering, nutrition, pharmaceuticals, and biomedicine^18–21^. However, pure gelatin systems often exhibit poor mechanical strength and rapid degradation under physiological conditions. To overcome these drawbacks, blending gelatin with synthetic polymers such as polyvinylpyrrolidone has proven highly effective.

 Polyvinylpyrrolidone is a highly hydrophilic synthetic polymer renowned for its nontoxic and biocompatible nature. These favorable biological properties, combined with its exceptional chemical versatility, make it a widely utilized component in the fabrication of nanogels. Its structural backbone contains carbonyl groups capable of forming hydrogen bonds and intermolecular interactions with drug molecules and interacting with other polymers, which enhance the stability of polymeric networks and facilitate efficient drug encapsulation. PVP exhibits excellent solubility in solvents of varying polarities, strong binding properties, and effective stabilization of suspensions and emulsions, making it valuable in pharmaceutical and industrial applications, making it valuable in pharmaceutical and industrial applications^22^. In nanoparticle synthesis, PVP is widely employed to control particle size, shape, and dispersion by preventing aggregation during preparation^23,24^. Additionally, PVP is extensively utilized to increase the solubility and bioavailability of poorly water-soluble drugs by forming molecular adducts that inhibit crystallization and promote amorphous solid dispersions^25^. Furthermore, PVP plays a crucial role in sustained-release drug delivery systems^26^. Its amphiphilic nature enables interactions with both hydrophilic and hydrophobic compounds, broadening its utility.

The combination of gelatin and PVP yields stable polymeric materials such as hydrogels^27–29^ and nanogels^30^, distinguished by enhanced swelling behavior, improved structural integrity, and superior drug-loading capacity. The complementary attributes of gelatin and PVP broaden biomedical applications, particularly by improving the encapsulation efficiency, dissolution, and bioavailability of poorly water-soluble therapeutics. One of the most important poorly soluble drugs is silibinin.

  Silibinin is the major active constituent of milk thistle (*Silybum marianum*) seeds, has been extensively investigated for its therapeutic potential. has demonstrated remarkable efficacy in managing viral hepatitis, alcohol-induced fatty liver, and toxin-related hepatic injury^31,32^, as well as its role as a complementary therapy for chronic inflammatory liver disorders^33^. Beyond its hepatoprotective role, SB exhibits pronounced anticancer activity, with documented inhibitory effects against malignancies of the liver, prostate, colon, breast, and skin^34–38^. Despite its broad pharmacological potential, the clinical application of SB is limited due to its poor aqueous solubility (0.092 mg/mL) and low oral bioavailability (23–47%)^39,40^. SB is widely studied for its anticancer and hepatoprotective properties, but is limited by extremely low aqueous solubility and poor oral bioavailability. Therefore, encapsulating SB into pH-sensitive nanogel systems represents an effective strategy to enhance its solubility, stability, and controlled release behavior.

This study aimed to develop pH-responsive gelatin/PVP nanogels prepared by γ-irradiation for the controlled delivery of SB. To enhance solubility and dissolution, SB solid dispersions with γ-irradiated PVP were first formulated. γ-irradiation of PVP induces controlled chain scission and the formation of reactive sites (free radicals, oxidized groups), which can enhance the interaction between PVP and SB during solid dispersion formation, promoting amorphization and inhibiting recrystallization. A smart Gel/PVP nanogel was then synthesized via γ-irradiation polymerization and subsequently loaded with SB/PVP in the presence of NHS, yielding a pH-responsive nanogel system designed to improve bioavailability and enable site-specific delivery. The SB/Gel/PVP nanogel was systematically evaluated by in vitro drug release using the dialysis diffusion method under varying pH conditions, anticancer activity against HepG2 cell proliferation via MTT assay, and biocompatibility assessment through biochemical and histopathological analyses of liver and kidney tissues in rats treated with either free SB or SB/Gel/PVP at a dose of 25 mg/kg.

## Materials and methods

### Materials

Silibinin, polyvinylpyrrolidone (k90), N, N’-methylenebisacrylamide, and N-hydroxysuccinimide were procured from Sigma-Aldrich Co. (St Louis, MO, USA). Gelatin (  type B) was obtained from El Goumhoria Co. (Egypt). All other chemicals and reagents were acquired from Merck Limited (Mumbai, India) and were of analytical quality.

### Gamma irradiation of PVP

PVP was subjected to γ-irradiation at a dose of 20 kGy (dose rate 0.53 kGy/h) using a Cobalt-60 (^60^Co) source installed at the National Center for Radiation Research and Technology (NCRRT), Egyptian Atomic Energy Authority (EAEA). Before irradiation, PVP samples were sealed in nitrogen-packed bags to prevent radiation-induced oxidation of PVP^41^.

### Preparation of SB/PVP solid dispersions

Solid dispersions of SB with PVP were prepared using the solvent evaporation technique. Briefly, 0.1 g of SB was dissolved in 20 mL of methanol to obtain a clear solution, while 2 g of irradiated PVP powder was separately dissolved in 20 mL of methanol. The two solutions were then combined and stirred for 30 min to ensure uniform mixing. A polymer-to-drug ratio (PVP: SB) of 20: 1 (w/w) was maintained to promote optimal dispersion of SB within the PVP matrix^42–44^. The resulting mixture was desiccated under vacuum to remove residual solvent, after which the dried SB/PVP solid dispersion was scraped and pulverized using a mortar and pestle for subsequent characterization^45^.

### Preparation of gel/PVP nanogels

Gel and PVP solutions were prepared separately by dissolving 0.5 wt% Gel and 1 wt% PVP in 1% HCl under continuous stirring at 60 °C for 1 h until complete dissolution. This is because gelatin requires acidic conditions (pH below its isoelectric point) to achieve complete dissolution and maintain a homogeneous solution with PVP. This low pH also protonates amino groups and enhances miscibility. The Gel/PVP solution (v/v) was obtained by adding the Gel solution to the PVP solution and stirring for an additional 1 h at 60 °C until homogeneity was achieved. Subsequently, 0.05 wt% N, N′-methylenebisacrylamide was incorporated into the mixture with continuous stirring for 1 h. Finally, the prepared Gel/PVP solution was exposed to γ-irradiation at a dose of 5 kGy (dose rate 0.53 kGy/h).

### Loading of SB into Gel/PVP nanogel

An appropriate amount of SB/PVP nanogel equivalent to 400 mg of SB was dissolved in 10 mL of 1% HCl solution and stirred for 2 h to obtain a homogeneous mixture. This solution was subsequently combined with the prepared Gel/PVP nanogel in the presence of 0.1 mol dm⁻³ NHS, which was employed to enhance the stability and functionality of the nanogel in biological buffer systems^44^. The SB/Gel/PVP nanogel was cast into sterile petri dishes and dried at ambient temperature, producing uniform solid films suitable for subsequent physicochemical characterization and biological evaluation.

The entrapment efficiency (%) of SB in Gel/PVP nanogels was determined using UV/Vis spectrophotometer (MicroDigital Co. Ltd., South Korea). First, the free unencapsulated drug was separated from the nanogel suspension through centrifugation, then the absorbance of the supernatant was measured at 288 nm against a standard calibration curve of SB solutions. The concentration of free SB is quantified, and the amount of entrapped SB is calculated by subtracting this value from the initial drug input. Finally, the entrapment efficiency (%) could be calculated using Eq. (1), and the loading capacity (%) of SB in Gel/PVP nanogels can be determined using Eq. (2).

1 $$\:{\mathrm{The}}\:{\mathrm{entrapment}}\:{\mathrm{efficiency}}\:\left( \% \right) = \frac{{{\mathrm{Abs}}\:{\mathrm{of}}\:{\mathrm{free}}\:{\mathrm{SB}} - \:{\mathrm{Abs}}\:{\mathrm{of}}\:{\mathrm{SB}}\:{\mathrm{in}}\:{\mathrm{supernatant}}}}{{{\mathrm{Abs}}\:{\mathrm{of}}\:{\mathrm{free}}\:{\mathrm{SB}}}}\: \times \:100$$

2 $$\:{\mathrm{The}}\:{\mathrm{loading}}\:{\mathrm{capacity}}\:\:\left( \% \right) = \frac{{{\mathrm{Entrapped}}\:{\mathrm{drug}}\:{\mathrm{amount}}}}{{{\mathrm{Total}}\:{\mathrm{nanogel}}\:{\mathrm{mass}}}}\: \times \:100$$

### Characterization analysis

#### Fourier transform infrared spectroscopy

FTIR spectra of SB, Gel, PVP, Gel/PVP, SB/PVP, and SB/Gel/PVP nanogels were recorded in the range of 500–4000 cm⁻¹ using a VERTEX 70 spectrometer (Bruker Optics Inc., Ettlingen, Germany). The analysis was performed to verify the successful incorporation and interaction of all components within the nanogel system, as evidence of functional group integration^9^.

#### X-ray diffraction

X-ray diffraction (XRD) analysis was performed using a Shimadzu XRD-6000 series diffractometer (Kyoto, Japan) to investigate the XRD characteristics of the Gel, PVP, Gel/PVP, SB, SB/PVP nanogel, and the SB/Gel/PVP composite^46^.

#### Transmission electron microscopy

The particle size and morphology of SB/Gel/PVP nanogels were examined using transmission electron microscopy (TEM, JEOL JEM-100CS, Japan). Samples were prepared by dispersing the nanogels in distilled water, placing a drop of the suspension onto a carbon-coated copper grid, and allowing it to dry under ambient conditions ^47,48^.

#### Dynamic light scattering

Dynamic light scattering (DLS) was employed to determine the average hydrodynamic particle size of SB/Gel/PVP nanogels using a DLS-ZP/Particle Sizer (NICOMP 380 ZLS, USA). Measurements were conducted under neutral conditions and in buffer solutions at pH 1.2, 4.5, 5.5, 6.5, and 7.4 to evaluate the pH-responsive behavior of the nanogels. For each measurement, SB/Gel/PVP was dispersed in 20 mL of distilled water or buffer, homogenized by ultrasonic treatment for 30 min, and subsequently filtered through a 0.45 μm membrane to remove large aggregates. The resulting supernatant was then used for particle size analysis, ensuring accurate and reproducible determination of hydrodynamic diameters^48^.

#### Measurement of the zero-point charge

The zero point of charge (pHzcp) of the SB/Gel/PVP nanogel was determined using the potentiometric mass titration method in 0.1 M NaCl aqueous solutions across a range of pH values ^49^. Initial pH readings were recorded for each solution, after which 50 mL was mixed with 0.1 g of SB/Gel/PVP nanogel and equilibrated for 24 h. The pH of the supernatant was then measured, and the pHzcp was calculated by applying linear regression to plots of initial pH versus final pH and initial pH versus ΔpH, thereby identifying the point at which the nanogel surface charge becomes neutral.

### In vitro drug release

The in vitro drug release of SB-loaded nanogels was evaluated using the dialysis diffusion method^50^. Before use, dialysis tubes were pre-soaked in PBS (pH 7.4) for 24 h and rinsed with distilled water. Unprocessed SB (10 mg) and an equivalent dose of SB/Gel/PVP nanogel were then placed into separate tubes, which were immersed in 200 mL of dissolution medium adjusted to pH 1.2, 4.5, 5.5, 6.8, or 7.4. The system was maintained at 37 °C under continuous stirring (100 rpm) to ensure uniform drug distribution. At predetermined intervals, 5 mL aliquots were withdrawn and replaced with equal volumes of fresh medium. SB concentrations were quantified by measuring absorbance at 288 nm using a UV/Vis spectrophotometer (MicroDigital Co. Ltd., South Korea), and cumulative release percentages of SB/Gel/PVP nanogels were calculated according to Eq. (3).

3 $$\:Drug\:released\:\left( \% \right) = \frac{{amount\:of\:drug\:released}}{{amount\:of\:loaded\:drug\:}} \times \:100$$

### Anticancer efficacy of SB/Gel/PVP nanogel

#### Cell culture

The human hepatocellular carcinoma (HCC) cell line HepG2 (ATCC^®^ HB-8065™) was originally sourced from the American Type Culture Collection (ATCC, Manassas, VA, USA) and generously provided by Dr. Ali M. (CSEIV, National Research Center, Dokki, Egypt). HepG2 cells were cultured in Dulbecco’s Modified Eagle’s Medium (DMEM; Lonza, Walkersville, USA) supplemented with 10% fetal bovine serum, 100 mg/mL streptomycin, and 100 U/mL penicillin (Gibco, Grand Island, NY)^51^. Subsequently, the cells were maintained at 37 °C in a humidified atmosphere containing 5% CO₂ within a Class II biological safety cabinet incubator.

#### In vitro anticancer activity of SB/Gel/PVP (MTT assay)

The anticancer efficacy of SB/Gel/PVP nanogel against HepG2 cells was evaluated using the MTT assay. Briefly, 96-well plates were seeded with 1 × 10⁴ cells per well and incubated for 24 h before treatment. Pure SB and SB/Gel/PVP were added to the media at various concentrations of SB (7.8, 15.6, 31.25, 62.5, and 125 µg/mL). For the SB/Gel/PVP nanogel, the added amount was calculated based on the drug loading content to ensure that the stated concentrations represent the equivalent concentration of SB, not the total weight of the nanogel. Next, the cells were incubated for 48 h. The cells that were not exposed to drugs served as the control group. Then, the media containing SB and SB/Gel/PVP were carefully removed, and 200 µL of 2 mg/ml MTT (3-(4,5-dimethylthiazol-2-yl)- 2,5-diphenyltetrazolium bromide) (Serva Electrophoresis GmbH, Germany) was added to each well. The plates were then incubated at 37 °C for 4 h. Afterward, all the contents of the wells were carefully removed, and 200 µL of DMSO was added to each well to dissolve the formazan crystals^52^. Finally, the optical density (OD) was recorded at λmax 570 nm using 96-well microtiter plates (Greiner Bio-One, Germany). The experiment was conducted three times, and the following Eq. (4) was adopted to evaluate the cell viability:

4 $$\:Cell\:viability\:\% = \frac{{OD\:at\:570\:nm\:of\:treated\:cells}}{{OD\:at\:570\:nm\:of\:untreated\:cells}}\: \times \:100$$

The IC_50_ was calculated using OriginPro software version 2023 (OriginLab Corp., Northampton, MA, USA.) by performing a non-linear regression (sigmoidal dose-response fit). (1) Cell viability percentages (Y) were normalized so that untreated controls represented 100% viability and background represented 0%. (2) SB concentrations (X) were log-transformed ($\:{\mathrm{Log}}_{10}\mathrm{Concentration}$). (3) Normalized viability data were plotted against the logarithm of concentration.

(4) The built-in DoseResp function was applied to fit the experimental data using the four-parameter logistic (4PL) Eq. (5)

5 $$\:Y = A1 + \frac{{A2 - A1}}{{1 + 10^{{\left( {LogX_{0} - X} \right)P}} }}$$

Where $\:{A}_{1}$ is the bottom plateau (0% viability), $\:{A}_{2}$ is the top plateau (100% viability), $\:Log{X}_{0}$ is the Log of IC_50_, and $\:P$ is the Hill slope.

From the fitted curve, OriginPro directly reported the Log(IC_50_) value (parameter $\:\mathrm{log}{X}_{0}$). The final IC_50_ was then calculated following Eq. (6):

6 $$\:IC_{{50}} = \:10\:^{{\left( {Log\:X_{0} } \right)}}$$

The goodness of fit was assessed using the *R²* value and the 95% confidence interval of the IC_50_.

### In vivo biocompatibility

#### Experimental design

Healthy male Wistar albino rats (180–220 g) were obtained from the National Center for Radiation Research and Technology, Egyptian Atomic Energy Authority, Egypt. The animals were housed under standard laboratory conditions with unrestricted access to food and water. All animal experiments were conducted in strict accordance with the National Institutes of Health Guide for the Care and Use of Laboratory Animals (8th Edition, 2011), the International Guiding Principles for Biomedical Research Involving Animals (CIOMS/ICLAS, 2012), and the ARRIVE 2.0 guidelines. The principles of Replacement, Reduction, and Refinement (3Rs) were rigorously applied throughout the study. Animal euthanasia procedures followed the AVMA Guidelines for the Euthanasia of Animals (2020 Edition). The protocol for animal care and handling was reviewed and approved by the Animal Care and Use Committee of the Zoology Department, Faculty of Science, Benha University, Egypt (ZD/FSc/BU-IACUC/2023-23b). Eighteen rats were randomly allocated into three groups (*n* = 6). The control group received saline by oral gavage, while the treatment groups were administered either free SB or SB-loaded Gel/PVP nanogel, both delivering SB at a dose of 25 mg/kg via oral gavage for 14 consecutive days^53^.

#### Evaluation of biochemical parameters

At the end of the experimental period, rats were euthanized by intraperitoneal injection of urethane (1.5 g/kg)^54^. Blood samples were collected under anesthesia and allowed to clot for 1 h at room temperature without anticoagulants. The samples were then centrifuged at 14,000 rpm for 20 min, and the resulting sera were stored at − 20 °C until analysis. Serum alanine transaminase (ALT), aspartate transaminase (AST), albumin, total bilirubin, urea, uric acid, and creatinine were quantified using colorimetric assay kits (Spectrum Diagnostics, Egypt) according to the manufacturer’s instructions.

#### Histopathological studies

At the end of the experimental period, liver and kidney tissue specimens were collected for histological examination. Samples were immediately fixed in 10% neutral buffered formalin for 72 h. Following fixation, tissues were dehydrated through a graded ethanol series, cleared in xylene, and embedded in paraffin wax. Paraffin blocks were sectioned at approximately 5 μm thickness using a rotary microtome. Sections were stained with hematoxylin and eosin (H&E) according to standard protocols^55^. Histological evaluation was performed using a Nikon Eclipse E800 light microscope, and representative photomicrographs were captured with an Olympus digital camera.

### Statistics

Statistical analyses were performed by SPSS version 25.0 (IBM Corp., Armonk, NY, USA). The graphs were generated by OriginPro version 2023 (OriginLab Corp., Northampton, MA, USA.). The values are presented as the mean ± standard deviation. One-way ANOVA was performed, followed by the LSD post hoc test, to determine statistically significant differences between the groups. Statistical significance was defined as *P* < 0.05.

## Results and discussion

The formulation of SB-loaded Gel/PVP nanogels was systematically optimized by varying polymer ratios to achieve desirable physicochemical properties. The selected polymer concentrations (0.5 wt% Gel and 1 wt% PVP) were chosen to ensure complete dissolution, optimal viscosity, and the formation of a stable polymeric network capable of efficient drug encapsulation. A γ-irradiation dose of 5 kGy was applied as it provides sufficient crosslinking to stabilize the nanogel structure while minimizing polymer degradation. The polymer-to-drug ratio of 20:1 (w/w) was maintained to maximize encapsulation efficiency and sustain controlled release without compromising nanogel integrity. Furthermore, NHS was incorporated to enhance chemical stabilization by promoting covalent interactions between SB and the polymer matrix, thereby improving drug loading, bioavailability, and pH-responsive release behavior. The optimized formulation demonstrated that polymer composition strongly influenced nanogel integrity, drug loading, and pH-dependent release behavior, thereby underscoring the importance of ratio selection in developing an effective delivery system. Concurrently, this synergistic interaction improved coupling efficiency, crosslink density, and stability in physiological buffers.

### FTIR results

**Fig. 1:**
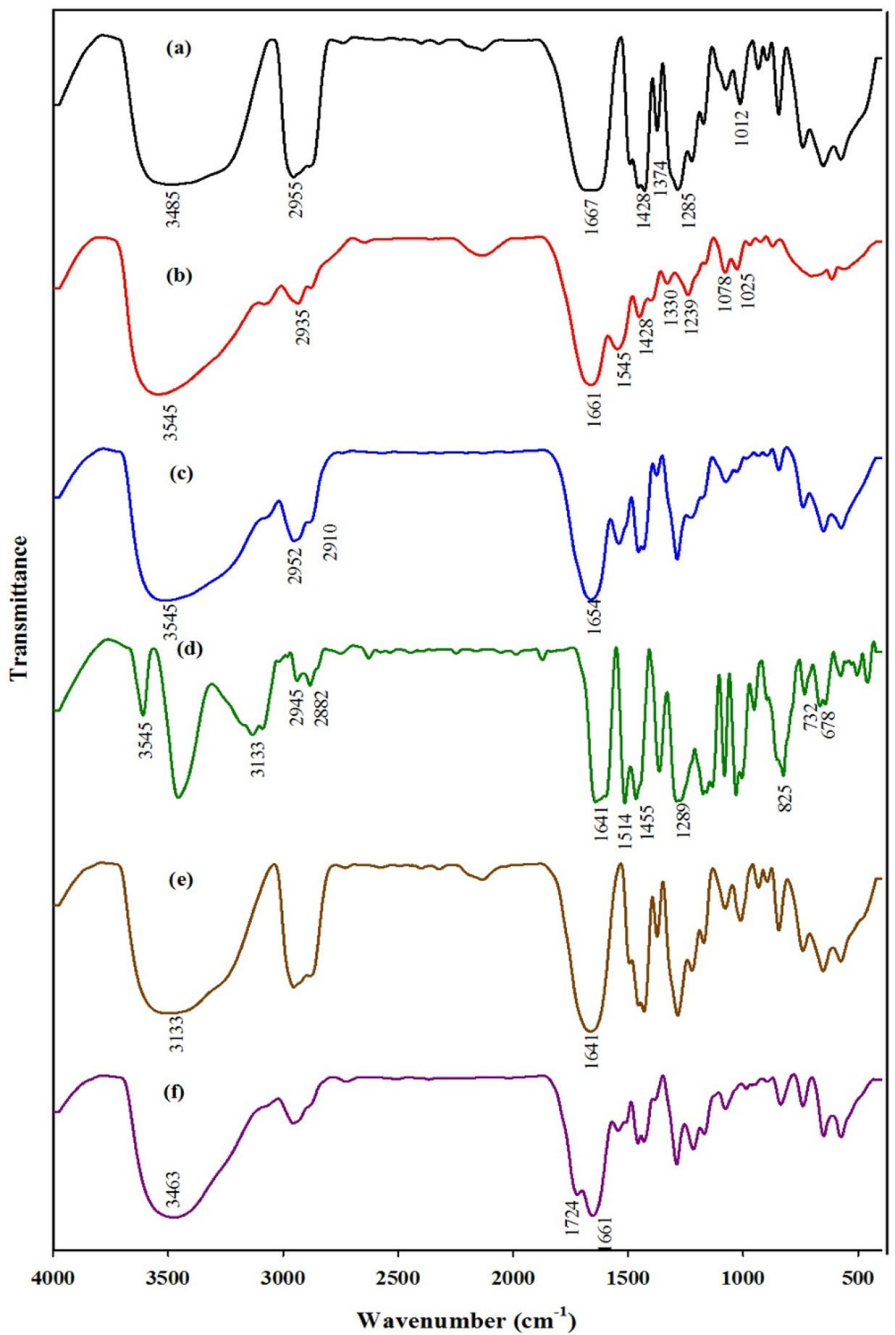
FTIR spectra of (**a**) PVP, (**b**) Gel, (**c**) Gel/PVP nanogel, (**d**) SB, (**e**) SB/PVP nanogel, and (**f**) SB/Gel/PVP nanogel.

Figure 1 presents the FTIR spectra of SB, PVP, Gel, Gel/PVP nanogel, SB/PVP, and the SB/Gel/PVP nanogel, enabling identification of functional groups and confirmation of the chemical structures. Figure 1a shows the FTIR spectrum of PVP, characterized by distinct absorption peaks: a broad peak at 3485 cm⁻¹ corresponding to O–H stretching vibrations of adsorbed water, a peak at 2955 cm⁻¹ attributed to C–H stretching, and a strong peak at 1667 cm⁻¹ assigned to the carbonyl (C = O) stretching vibration. Additional peaks at 1428 and 1374 cm⁻¹ are associated with pyrrolidone ring vibrations, while peaks at 1285 cm⁻¹ and 1012 cm⁻¹ correspond to C–N stretching and CH₂ rocking, respectively^56,57^.

Figure 1b presents the FTIR spectrum of Gel, which exhibits characteristic absorption peaks at 3545 cm⁻¹ (N–H stretching) and 2935 cm⁻¹ (aliphatic C–H stretching). The amide I peak at 1661 cm⁻¹ arises from C = O stretching, while the amide II peak at 1545 cm⁻¹ corresponds to N–H bending coupled with C–N stretching. The amide III peak at 1239 cm⁻¹ is attributed to N–H bending and C–H stretching vibrations^58^. A peak at 1661 cm⁻¹ also reflects asymmetric COO⁻ stretching, whereas peaks at 1078 cm⁻¹ and 1025 cm⁻¹ are due to C–N stretching. Peaks in the range of 1451–1330 cm⁻¹ are assigned to symmetric and asymmetric bending vibrations of methyl (CH₃) groups^59^.

Figure 1c shows the FTIR spectrum of the Gel/PVP nanogel, which displays the characteristic absorption peaks of both PVP and Gel. Asymmetric and symmetric C–H stretching vibrations were observed at 2952 and 2910 cm⁻¹, respectively. A peak at 3515 cm⁻¹ corresponds to O–H and N–H stretching vibrations. The C = O stretching peak of PVP, originally at 1667 cm⁻¹, shifted to 1654 cm⁻¹ in the nanogel, confirming hydrogen bond formation between the carbonyl groups of PVP and hydroxyl groups of Gel. These spectral changes provide evidence of successful copolymerization and intermolecular interactions between PVP and Gel^27^. Figure 1d presents the FTIR spectrum of SB, which exhibits characteristic peaks at 3509–3456 cm⁻¹ assigned to O–H stretching vibrations. The peak at 3133 cm⁻¹ corresponds to aromatic C–H stretching, while peaks in the range of 2945–2882 cm⁻¹ are attributed to aliphatic C–H stretching. A strong absorption at 1641 cm⁻¹ is due to C = O stretching, whereas peaks at 1514–1455 cm⁻¹ reflect aromatic C = C skeletal vibrations. The peak at 1289 cm⁻¹ is assigned to C–O–C stretching. Additional peaks at 825, 732, and 678 cm⁻¹ correspond to C–H bending vibrations of the benzene ring^37^.

Figure 1e shows the FTIR spectrum of the SB/PVP nanogel, which exhibits a broad peak at 3494 cm⁻¹ attributed to O–H and N–H stretching vibrations. A distinct peak at 1641 cm⁻¹ corresponds to C = O stretching, while the typical vibrational peaks of SB remain visible, confirming its presence within the nanogel .

Figure 1f presents the FTIR spectrum of the SB/Gel/PVP nanogel, which combines the characteristic peaks of PVP, Gel, and SB with notable spectral shifts due to chemical interactions among the three components. The O–H and N–H stretching peak shifted to 3463 cm⁻¹, reflecting hydrogen bond formation. The C = O stretching peaks originally observed at 1667 cm⁻¹ (PVP), 1661 cm⁻¹ (Gel), and 1641 cm⁻¹ (SB) collectively shifted to 1653 cm⁻¹, accompanied by the emergence of a new peak at 1724 cm⁻¹ due to the chemical interaction between the hydroxyl groups of SB and the carboxyl groups of gelatin/PVP. This peak does not appear in the spectra of pure PVP, pure gelatin, or Gel/PVP nanogel supporting their interaction. These spectral changes confirm the successful incorporation of SB into the Gel/PVP copolymer matrix and highlight the formation of a stable nanogel network.

### XRD results

**Fig. 2:**
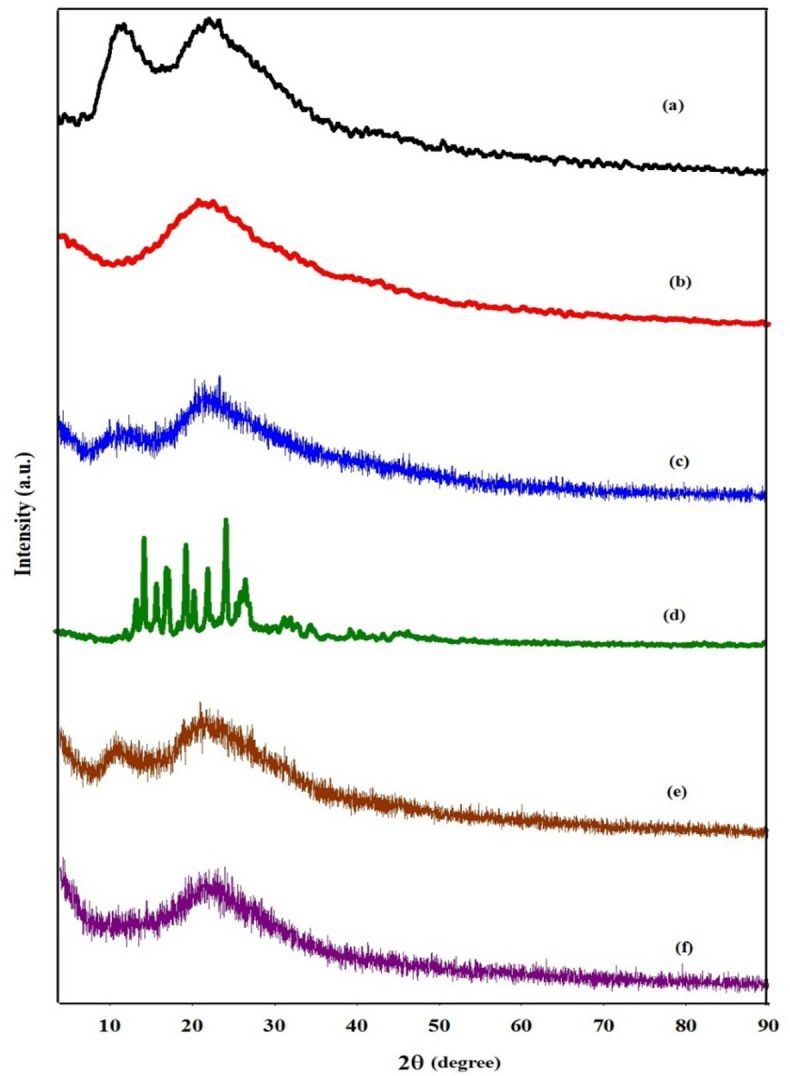
XRD patterns of (**a**) PVP, (**b**) Gel, (**c**) Gel/PVP nanogel, (**d**) SB, (**e**) SB/PVP nanogel, and (**f**) SB/Gel/PVP nanogel.

Figure 2 shows the XRD patterns used to investigate the crystalline and amorphous characteristics of the studied materials. Figure 2a shows the XRD pattern of PVP, characterized by two broad diffraction peaks at 2θ = 11.4° and 22.1°^27^, confirming its semi-amorphous nature. Figure 2b presents the XRD curve of Gel, which displays a single broad peak at 2θ ≈ 21.6°, indicative of its amorphous structure^60^. The Gel/PVP nanogel (Fig. 2c) exhibits two peaks; a weak reflection at 2θ = 11.6° and a broad peak at 2θ = 21.5°—suggesting intermolecular interactions between PVP and Gel chains. Figure 2d shows the XRD pattern of SB, which reveals multiple sharp peaks at 2θ values of 13.77°, 14.4°, 14.7°, 16.2°, 17.4°, 17.7°, 19.8°, 20.8°, 22.5°, 24.3°, 24.7°, 25.9°, 26.5°, 27.0°, 27.5°, 32.5°, 34.9°, 39.7°, and 43.2°, confirming its crystalline nature^61^. The SB/PVP nanogel (Fig. 2e) displays two major diffraction peaks at 2θ = 10.8° and 21.8°, with reduced intensity and slight shifts compared to pure PVP, indicating amorphous dispersion due to SB–PVP interactions. Finally, the SB/Gel/PVP nanogel (Fig. 2f) shows a single broad peak centered at 2θ ≈ 22.1°, confirming the amorphous status of the composite nanogel.

### TEM, DLS, and surface charge of SB/Gel/PVP nanogel

**Fig. 3:**
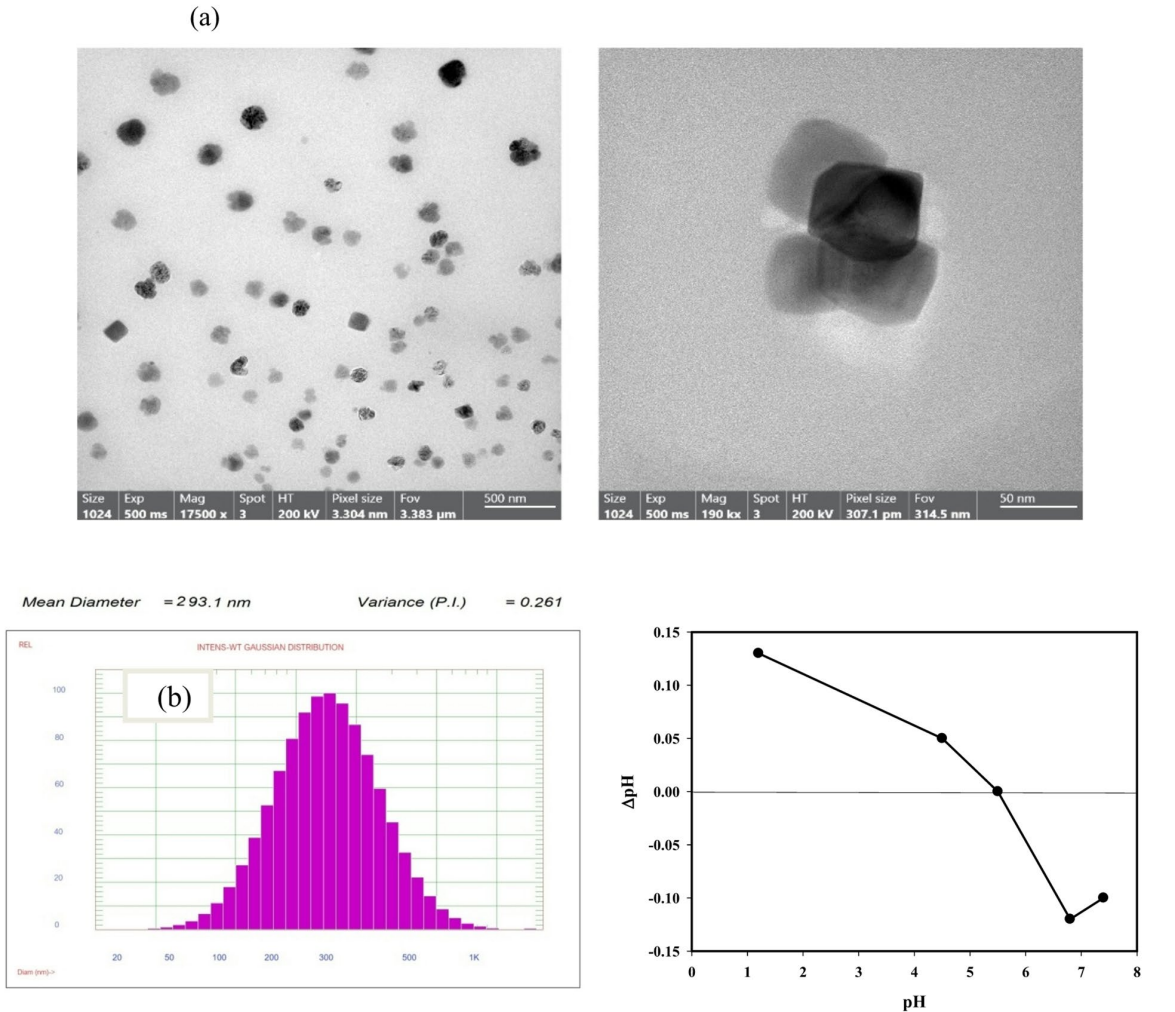
(**a**) TEM micrograph showing the morphology and particle size of SB/Gel/PVP nanogels, (**b**) DLS analysis indicating the hydrodynamic particle size distribution, and (**c**) surface charge behavior as a function of pH, highlighting the zero-point of charge (pHzcp).

Figure 3a presents TEM micrographs of SB/Gel/PVP nanogels, revealing a uniform distribution of highly dispersed, pseudo-cubic and irregular nanoparticles with a particle size below 50 nm. In the low-magnification image (scale bar 500 nm), the nanoparticles appeared relatively uniformly dispersed, and dark polyhedral particles were present. In the high-magnification image (scale bar 50 nm), a cluster of faceted nanoparticles is clearly visible, with sharp edges and overlapping cubic shapes that highlight their crystalline nature.

Figure 3b shows the mean particle size of the SB/Gel/PVP nanogels as determined by DLS. The nanogels exhibited an average hydrodynamic diameter of 293 nm, reflecting a uniform and homogeneous colloidal morphology. This value is larger than the dry particle size observed by TEM, which can be attributed to the swelling behavior of the nanogels in aqueous solution and their tendency to associate through intermolecular forces such as van der Waals interactions and hydrogen bonding. Despite this, colloidal stability was maintained primarily through electrostatic repulsion between particles, which minimized aggregation and ensured effective dispersion in suspension^62^. This difference arises because TEM provides the dry particle size under vacuum, while DLS measures the hydrodynamic diameter in aqueous suspension, which includes the hydration shell and the swelling of the nanogel network. The six-fold increase in size suggests that the nanogel undergoes substantial swelling in aqueous media.

Figure 3c depicts the pH-dependent variation in the surface charge of SB/Gel/PVP nanogel particles at various pHs of 1.2, 4.5, 5.5, 6.8, and 7.4. The term pHzcp refers to the pH at the zero-point of charge, which represents the condition at which the net surface charge of the nanogel becomes neutral. This parameter was determined using potentiometric mass titration, The nanogel exhibited positive surface charge at pH 1.2 and pH 4.5, and exhibited negative surface charge at pH 6.8 and 7.4, confirming its pH-sensitive behavior. The net charge approaches neutrality at pH ≈ 5.5, corresponding to the isoelectric point of the system; below this value, the nanogels carry a positive charge, whereas above it they become negatively charged. This charge reversal reflects the protonation and deprotonation of gelatin functional groups, where cationic –NH₃⁺ dominates under acidic conditions and anionic –COO⁻ under basic conditions. The overall results would support that the SB/Gel/PVP nanogel possesses pH-sensitive surface properties for controlled and stimuli-responsive drug delivery applications.

### Effect of pH on particle size of SB/Gel/PVP

**Fig. 4:**
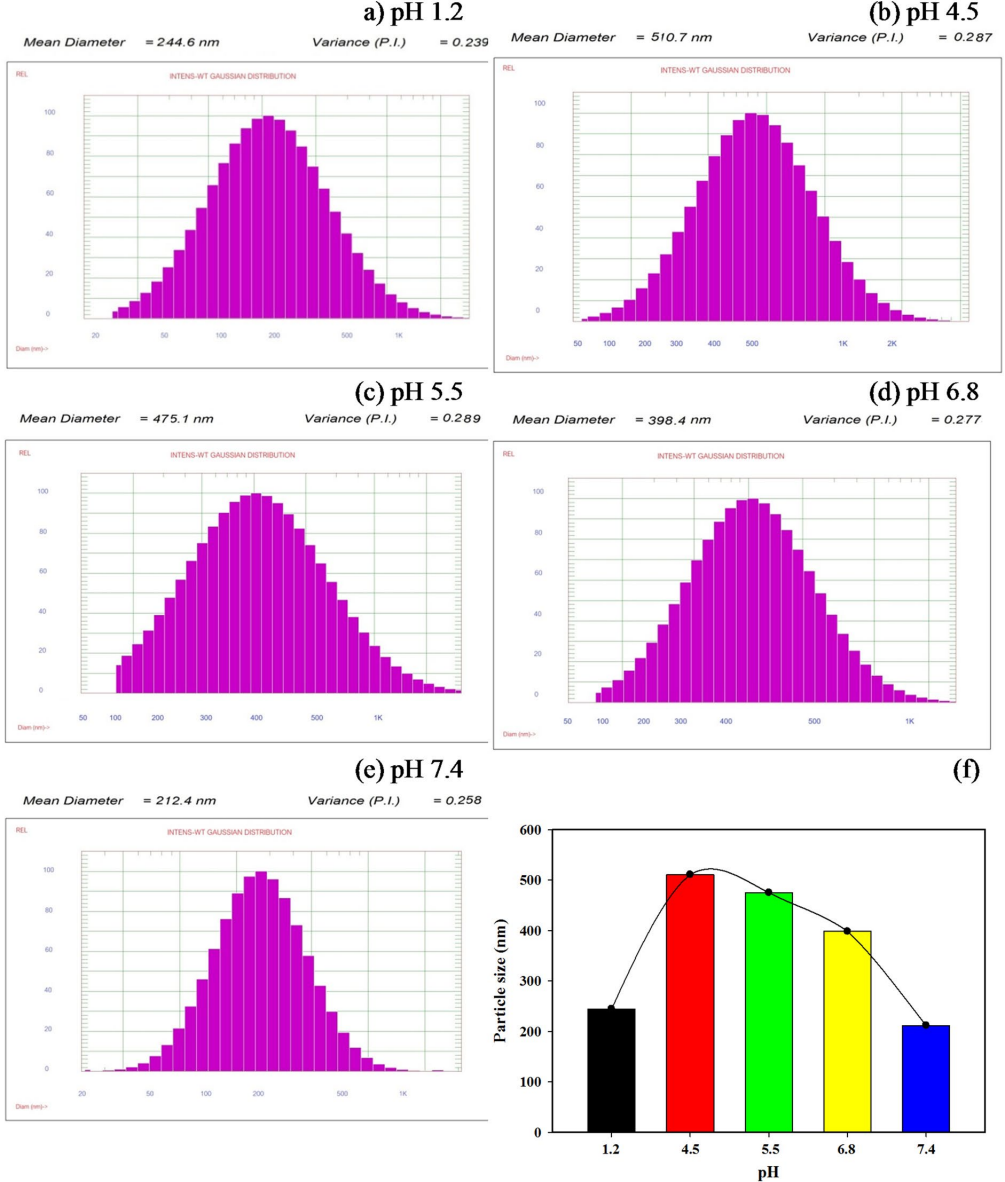
The average particle size from DLS measurements of SB/Gel/PVP at various pH values (**a**-**e**) and (**f**) relation between the particle size at different pHs.

Nanogels are nanoscale hydrogel particles characterized by their unique swelling behavior in response to environmental stimuli, particularly pH variations, which enables the controlled release of therapeutic agents under physiological conditions such as those found in tumor microenvironments. To evaluate this property, DLS analysis was performed to monitor changes in the particle size of SB/Gel/PVP nanogels as a function of pH. As shown in Fig. 4, the average particle size was measured at pH values of 1.2, 4.5, 5.5, 6.8, and 7.4, demonstrating the pH-responsive behavior of the system.

At pH 1.2 (gastric medium, Fig. 4a), the SB/Gel/PVP nanogels exhibited an average particle size of 244 nm. In contrast, larger sizes were observed at pH 4.5 (510 nm, Fig. 4b) and pH 5.5 (475 nm, Fig. 4c), which correspond to the acidic environment of intracellular endosomal and lysosomal compartments in cancer cells, indicating pronounced swelling. At pH 6.8 (intestinal medium, Fig. 4d), the nanogels exhibited a size of 398 nm, whereas at pH 7.4 (blood and normal cells, Fig. 4e), the size decreased markedly to 212 nm. The overall relationship between particle size and pH is summarized in Fig. 4f. These variations can be attributed to the swelling capacity and polyampholytic nature of gelatin ^63^, which contains ionizable functional groups such as carboxyl (–COOH), amino (–NH₂), and hydroxyl (–OH). Its amphoteric character allows structural and conformational changes in response to environmental stimuli, with its net surface charge varying according to pH. Under highly acidic conditions (pH 1.2), the abundance of free hydrogen ions keeps most carboxyl groups unionized, promoting hydrogen bonding between –COOH and –OH groups, thereby condensing the polymeric network and reducing particle size ^64^. As the pH increases to 4.5–5.5, ionization of carboxylate groups (–COO⁻) induces swelling, reflected in the larger particle sizes. At higher pH values (6.8–7.4),–COO⁻, –NH₂, and –OH groups are fully deprotonated by the charge screening effect or ‘salting-out’ effect of Na⁺ counterions present in the buffer solution, leading to a reduction in the hydrodynamic diameter of the nanogel.

The SB/Gel/PVP nanogels consistently exhibited polydispersity index (PI) values of 0.239, 0.287, 0.289, 0.277, and 0.258 at pH 1.2, 4.5, 5.5, 6.8, and 7.4, respectively. These values confirm that the nanogels are essentially monodisperse, demonstrating uniform colloidal stability with minimal aggregation and supporting the conclusion that the system maintains pharmaceutical-grade dispersity and stability.

Moreover, Fig. 4f showed a marked reduction in hydrodynamic diameter, especially at pH 7.4, which is primarily due to the high ionic strength of the phosphate buffer, and the presence of sodium ions in the buffer partially neutralizes these charges by electrostatic screening. This reduces the effective repulsion between polymer chains, leading to a more compact nanogel network. Additionally, the gelatin type can influence pH responsiveness. The gelatin used in our formulation was Type B, which has an isoelectric point of approximately pH 4.8–5.2. At pH 7.4, Type B gelatin carries a net negative charge, which would normally favor swelling; however, the ionic strength of the buffer overrides this effect and induces shrinkage.

### The entrapment efficiency and loading percentage of SB in Gel/PVP nanogel

The entrapment efficiency and loading capacity percentage of SB within the Gel/PVP nanogel were determined to be 82% and 3.34%, respectively. These values indicate that the majority of the initially introduced SB was successfully encapsulated within the nanogel matrix. These values that lie within the acceptable pharmaceutical range confirm the effective incorporation of SB into the copolymer matrix. This high efficiency is primarily attributed to strong hydrogen bonding interactions between SB and the carbonyl groups of PVP, as well as the polyelectrolytic nature of gelatin, which facilitates additional drug–polymer interactions through its ionizable functional groups. The finding contributes to the stable encapsulation of SB within the nanogel system, underscoring its suitability as a pH-responsive carrier for enhanced drug delivery. This result is consistent with other nanocarrier systems designed for poorly soluble SB^65,66^.

### Effect of pH on drug release

**Fig. 5:**
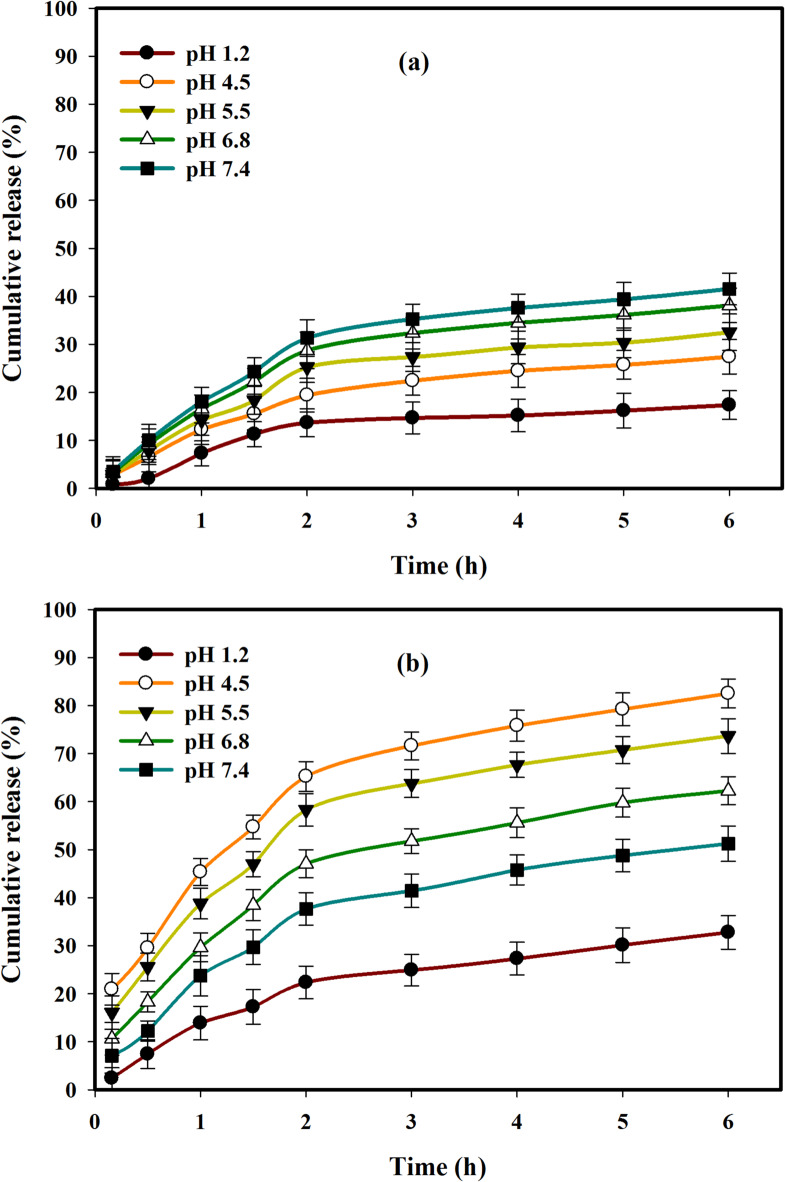
In vitro release of (**a**) free SB and (**b**) SB/Gel/PVP nanogel at different pH values.

One of the major challenges in drug delivery is achieving efficient release at the target site, particularly for poorly water-soluble drugs such as SB, whose solubility and dissolution are highly dependent on pH. Figure 5 depicts the in vitro release profiles of free SB and SB-loaded Gel/PVP nanogels over 6 h under different pH conditions. While the 6 h duration exceeds the typical gastric residence time (pH 1.2), the extended release profile was investigated to comprehensively characterize the pH-dependent swelling and diffusion behavior of the nanogel across all physiologically relevant pH environments, including intracellular endosomal/lysosomal compartments of cancer cells (pH 4.5–5.5), intestinal fluid (pH 6.8), and systemic circulation (pH 7.4).

As shown in Fig. 5a, pure SB exhibited a modest initial release within the first 2 h, followed by a slow and sustained release up to 6 h. At low pH values (1.2 and 4.5), the release was markedly suppressed, with cumulative release limited to approximately 15–25% after 6 h. This reduced release under acidic conditions can be attributed to the weakly acidic nature of SB, which remains predominantly in its non-ionized, poorly soluble form at low pH^67^. Such behavior is advantageous for oral delivery, as it minimizes drug degradation in the stomach and mitigates gastric irritation. At pH 5.5, the cumulative release of SB reached 38.5% after 6 h, representing an intermediate rate between gastric and intestinal conditions. A modest but progressive release was also observed at pH 6.8 and 7.4, with cumulative values of 38% and 41.5%, respectively, after 6 h. This behavior is because SB exhibits pH-dependent ionization due to its phenolic hydroxyl groups (pKa ~6–7). At acidic pH (1.2, 4.5, 5.5), SB remains largely non-ionized and poorly soluble, leading to very low release. As pH increases toward 6.8 and 7.4, deprotonation enhances aqueous solubility, resulting in the observed increase in cumulative release. However, even at pH 7.4, the solubility remains limited relative to the total drug load. These release profiles, corresponding to intestinal and systemic circulation media, reflect enhanced ionization and aqueous solubility of SB in less acidic to alkaline environments, primarily due to the deprotonation of phenolic hydroxyl groups. Such findings underscore the inherent limitations of unformulated SB in achieving therapeutic concentrations, particularly within acidic microenvironments such as tumors. To overcome these constraints, the present study focused on improving SB’s aqueous solubility through PVP-based solid dispersions and enhancing its release profile under acidic conditions via incorporation into a stimuli-responsive Gel/PVP nanogel system.

Figure 5b shows the cumulative release profile of SB from Gel/PVP nanogels under different pH conditions, revealing a pronounced pH-dependent pattern in two burst release stages of 2 h and 6 h. An initial burst release within the first 2 h, followed by a slower, sustained release phase extending to 6 h. The rapid initial release is attributed to loosely adsorbed or surface-associated SB/Gel/PVP complexes that dissolve quickly upon exposure to the release medium. After 2 h, the lowest release (22%) was observed at pH 1.2, simulating gastric conditions. At pH 4.5, markedly enhanced cumulative release of 65%, due to the pH of the medium becoming near the isoelectric point, thus the SB/Gel/PVP matrix undergoes conformational relaxation and partial ionization of carboxyl groups, facilitating drug diffusion. This behavior aligns with the isoelectric point of gelatin (typically pH 4.5–5), where high proton concentrations lead to protonation of gelatin’s functional groups, strengthening hydrogen bonding and electrostatic interactions that collapse the polymer network and restrict drug diffusion. Furthermore, amino acid residues within gelatin become protonated below their pKa (~ 3.5), increasing the net positive charge and reducing osmotic pressure inside the polymer matrix, thereby contributing to nanogel collapse and limited drug release^68^. At pH 5.5, the cumulative release was 58%, which is attributed to the increasing negative charge, that begins to induce partial electrostatic interaction with the partially ionized SB, slightly reducing the release percentage again. At pH 6.8 and 7.4, increased ionization of both the matrix and SB lead to stronger electrostatic interactions and charge screening effects, further reducing release to 47% and 37%, respectively.

After prolonged incubation (6 h), the second burst release, the cumulative release percentage increased, reflecting the controlled release of SB from the Gel/PVP nanogel matrix, with a similar trend observed under elevated pH conditions. The final cumulative release values were 33%, 82%, 73%, 62%, and 51% at pH 1.2, 4.5, 5.5, 6.8, and 7.4, respectively. These differences can be attributed to the varying degrees of ionization of reactive groups (–COOH and –NH₂) within the Gel/PVP nanogel under different pH conditions. The second release phase was characterized by controlled release of entrapped SB, confirming the nanogel’s capacity for prolonged delivery. The lowest release value was observed at a strongly acidic pH (1.2), which is attributed to the protonation of functional groups leading to network collapse and restricted diffusion. The highest release values observed at pH 4.5 (82%) and pH 5.5 (73%) can be attributed to the physicochemical behavior of both SB and the Gel/PVP nanogel matrix under moderately acidic conditions. At these pH values, partial ionization of gelatin carboxyl groups and deprotonation of SB’s phenolic hydroxyl groups promote nanogel swelling, loosening of the polymeric network, and enhanced drug diffusion. This swelling effect increases pore size and facilitates the release of encapsulated SB. Moreover, the highest release at pH 4.5 highlights the nanogel’s intelligent, stimuli-responsive behavior and its suitability for targeted drug delivery in acidic environments such as the tumor microenvironment. While at near-neutral pH (6.8–7.4), increased ionic strength and reduced swelling contribute to lower release percentages. These findings are consistent with previous reports demonstrating enhanced release of SB from nanoformulations under moderate acidic conditions^66,69,70^, reinforcing the potential of pH-sensitive carriers for tumor-targeted therapy. 

It is important to contextualize the observed pH-responsive release behavior within the physiological pH gradients relevant to cancer therapy. The extracellular pH of the tumor microenvironment is characteristically acidic, typically ranging from 6.0 to 6.8, due to the elevated glycolytic activity of cancer cells and the consequent accumulation of lactic acid, a phenomenon known as the Warburg effect^71^. This contrasts with the physiological pH of 7.4 in normal tissues and the bloodstream. In this regard, the substantial SB release observed at pH 5.5 (73%) and pH 4.5 (82%) corresponds to the pH encountered within intracellular endosomal and lysosomal compartments following cellular internalization of nanoparticles via endocytic pathways^72^. The enhanced drug release under these conditions suggests that, beyond extracellular tumor targeting at pH 6.0–6.8, the SB/Gel/PVP nanogel may facilitate efficient intracellular drug delivery upon uptake by cancer cells. This dual pH responsiveness, partial activation in the mildly acidic tumor interstitium (pH 5.5–6.8), and maximal release within the more acidic endolysosomal vesicles (pH 4.5–5.5), represents a therapeutically advantageous profile for sequential extracellular and intracellular drug delivery.

### In vitro anticancer efficiency of SB/Gel/PVP

**Fig. 6:**
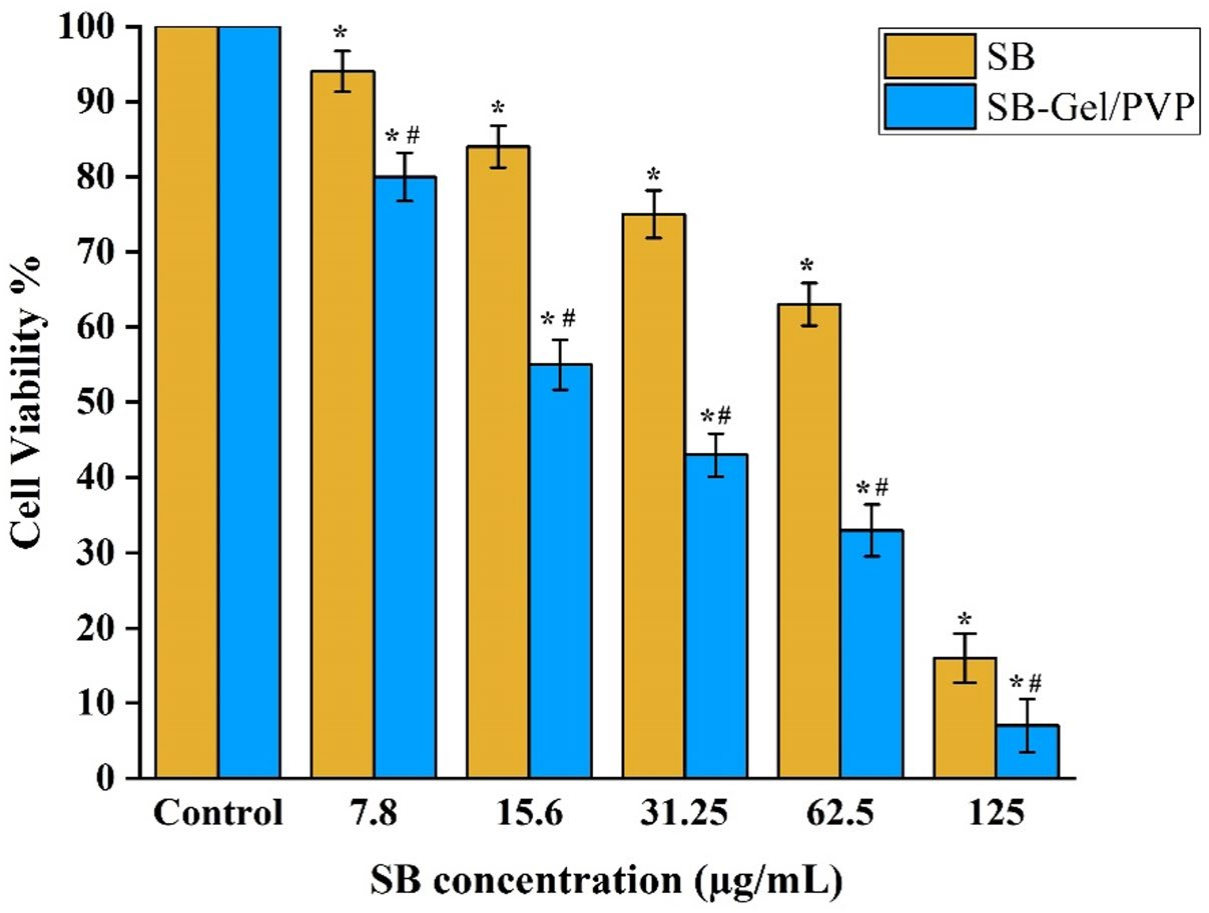
Impact of pure SB and SB/Gel/PVP on the growth of HepG2 cells. Data are presented as the mean ± SD from three distinct experiments. Significance is denoted by *p < 0.05 against the control group and ^#^*p* < 0.05 against the SB group.

Poorly water-soluble drugs often achieve only minimal plasma concentrations, insufficient to elicit therapeutic efficacy^73^. Approximately 40% of pharmaceutical compounds developed in the industry, including many anticancer agents, exhibit low cellular bioavailability due to limited aqueous solubility^74^. SB, despite its potent anticancer properties, faces similar challenges that restrict its clinical utility. To overcome this limitation, we developed a pH-responsive Gel/PVP nanogel system aimed at enhancing SB bioavailability and targeted delivery. The cytotoxic effects of free SB and SB-loaded Gel/PVP nanogel were assessed using the MTT assay in HepG2 HCC cells. As shown in Fig. 6, cell viability decreased significantly with increasing concentrations of both formulations (*P* < 0.05) (Supplementary Table S1). Notably, SB/Gel/PVP exhibited superior antiproliferative activity compared to free SB (*P* < 0.05), likely due to enhanced intracellular accumulation mediated by nanoparticle uptake. This increased potency was reflected in the IC_50_ values were 23.3 µg/mL for SB/Gel/PVP versus 66.6 µg/mL for free SB. Collectively, these findings demonstrate that the Gel/PVP nanogel effectively addresses the bioavailability barrier of SB, thereby amplifying its therapeutic potential.

Our findings are consistent with previous reports demonstrating improved SB delivery through nanocarrier systems. Tan et al.^75^ reported enhanced release and increased cytotoxicity of SB from a pH-responsive carbon nanotube-based system against multiple cancer cell lines compared to free SB. Gohulkumar et al.^61^ showed that encapsulating SB into Eudragit^®^ E nanoparticles stabilized with polyvinyl alcohol (PVA) significantly improved its anticancer efficacy in oral carcinoma (KB) cells. Kuen et al.^76^ developed hydrophobically modified chitosan nanoparticles (pCNPs), which enhanced SB’s cytotoxicity and reduced IC50 values in A549 lung cancer cells. In another study, Liu et al.^77^ formulated lipid–polymer hybrid nanoparticles modified with wheat germ agglutinin and coated with pHPMA, co-encapsulating SB and cryptotanshinone (S/C-W-LPNs). These hybrid nanoparticles markedly enhanced cytotoxicity against 4T1 breast cancer cells and effectively suppressed cell invasion and migration. Collectively, these studies underscore the transformative potential of nanoformulations in overcoming the physicochemical limitations of SB, particularly its poor solubility, dissolution rate, and in vitro release profile. In line with this evidence, our results confirm that the Gel/PVP nanogel not only improves drug delivery efficiency but also significantly augments the anticancer activity of SB.

It should be noted that in vivo antitumor efficacy was not assessed within the scope of the present study; thus, therapeutic claims remain confined to cellular-level observations. Notably, an in vivo evaluation of this formulation in a chemically induced HCC rat model has been conducted and reported separately^44^, providing complementary evidence of therapeutic potential beyond the scope of the current manuscript.

### Analysis of hepatic and renal functions

**Fig. 7:**
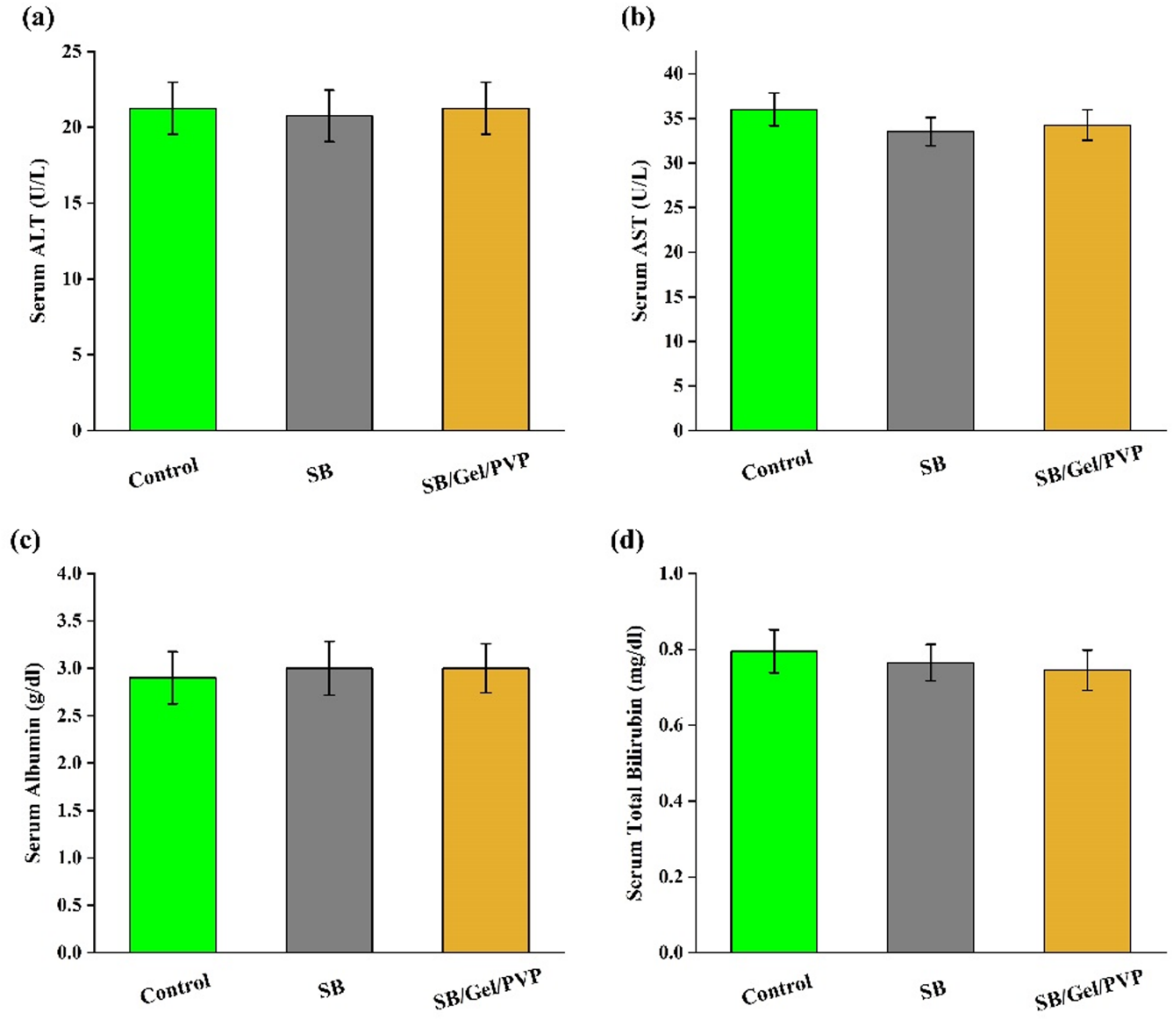
Effect of oral administration of SB and SB/Gel/PVP on (**a**) ALT, (**b**) AST, (**c**) albumin, and (**d**) total bilirubin in serum. Data are presented as mean ± SD. Liver biomarkers in the SB and SB/Gel/PVP groups exhibited minor differences from the control group (*P* > 0.05).

**Fig. 8:**
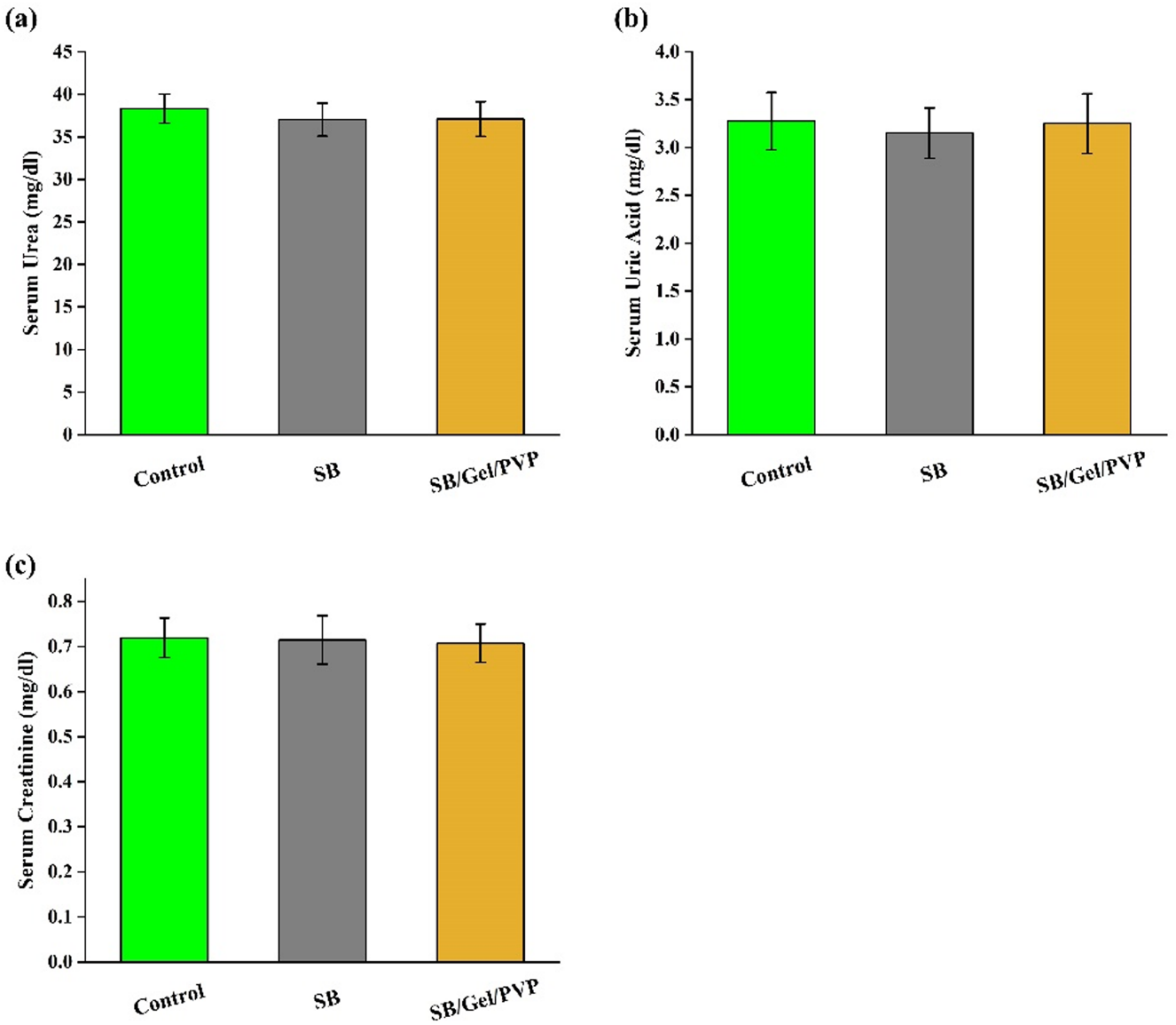
Impact of oral treatment with SB and SB/Gel/PVP on (**a**) urea, (**b**) uric acid, and (**c**) creatinine in serum. Data are reported as mean ± SD. Trivial differences in the renal biomarkers were observed in the SB and SB/Gel/PVP groups relative to the control group (*P* > 0.05).

The safety profile of SB-loaded nanogel was evaluated in vivo using male Wistar albino rats. Animals received oral administration of SB/Gel/PVP or free SB at a dose of 25 mg/kg for two weeks. Hepatic and renal biomarkers were measured in serum to assess potential adverse effects. As shown in Fig. 7, no significant differences were observed in liver function markers (ALT, AST, albumin, and total bilirubin) compared with the control group (*P* > 0.05). Similarly, minor deviations were detected in renal markers (urea, uric acid, and creatinine) in rats treated with SB-loaded nanogel or free SB relative to controls (Fig. 8, *P* > 0.05). These findings are consistent with previous reports^78–80^. Collectively, the data indicate that the SB/Gel/PVP nanogel is well-tolerated by both the liver and kidneys, with no evidence of significant histopathological or functional toxicity.

Nonetheless, a key limitation of this study is the absence of biodistribution and pharmacokinetic data. Without tracking the nanogel’s systemic fate, conclusions about target-site accumulation, clearance pathways, or long-term safety remain speculative. Radiolabeling or fluorescent tagging approaches will be employed in future work to address this gap.

### Histopathological examination

**Fig. 9:**
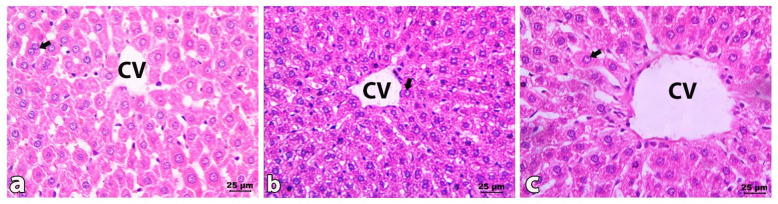
Representative photomicrographs of liver sections stained with H&E from (**a**) the control group, (**b**) the SB group, and (**c**) the SB/Gel/PVP group showing polygonal hepatocytes (arrows) with large spherical single nuclei arranged in typical strands around the central vein (CV).

Liver sections from the control group exhibited normal histoarchitecture, characterized by hexagonal lobules, polygonal hepatocytes arranged in trabeculae, a central vein, and peripheral hepatic triads (Fig. 9a). In the SB-treated group, liver sections displayed comparable features, with only mild central vein congestion and slight vacuolar degeneration in some hepatocytes (Fig. 9b). In the SB/Gel/PVP group, mild portal vein congestion and periportal vacuolation of hepatocytes were observed (Fig. 9c). These findings are consistent with previous reports^79–81^.

**Fig. 10:**
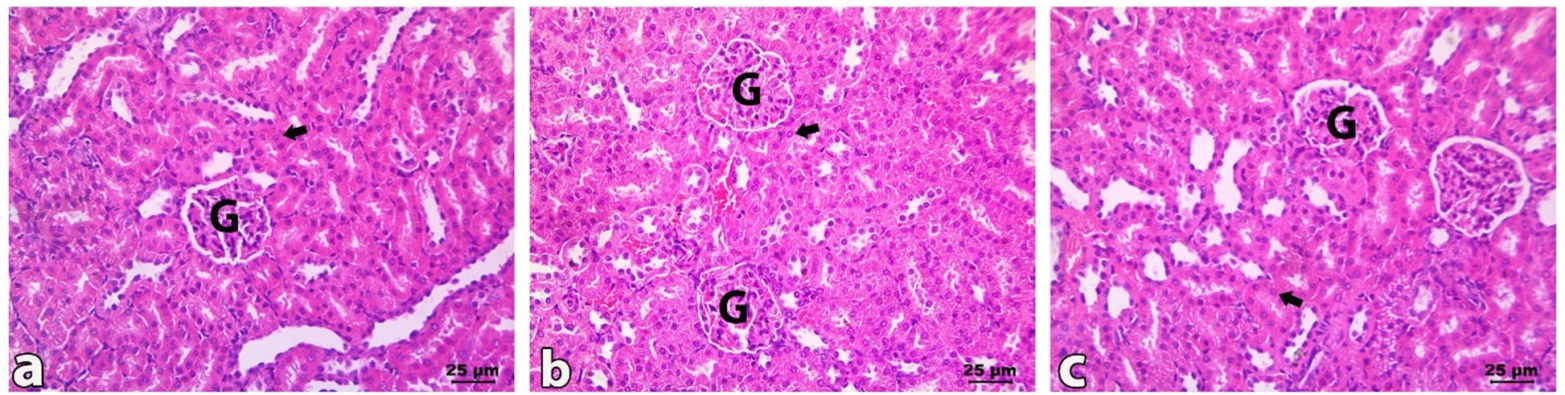
Representative photomicrographs of kidney sections stained with H&E from (**a**) the control group, (**b**) the SB group, and (**c**) the SB/Gel/PVP group showing renal corpuscles with normal glomeruli (G) and renal tubules lined with simple cuboidal epithelium (arrows).

Kidney sections from the control group exhibited normal histological architecture of the cortex and medulla. The renal corpuscles consisted of glomeruli enclosed by Bowman’s capsules, while the renal tubules were lined with a single layer of cuboidal cells displaying eosinophilic cytoplasm and rounded nuclei (Fig. 10a). In the SB-treated group, kidney sections showed a preserved microscopic appearance comparable to the control (Fig. 10b). Likewise, sections from the SB/Gel/PVP group demonstrated well-maintained histological integrity relative to the control (Fig. 10c). These observations are consistent with previous reports^82,83^. The preservation of intact renal histology, together with minimal alterations in hepatic and renal function parameters following SB/Gel/PVP treatment, supports favorable host–tissue compatibility. This biocompatibility is attributable to the intrinsic properties of gelatin and PVP, including their established biocompatibility and tunable degradation profiles^19,25,84,85^. Collectively, these characteristics position the Gel/PVP nanogel as a promising platform for sustained and targeted SB delivery.

## Conclusion

An oral pH-sensitive Gel/PVP nanogel was successfully synthesized via gamma-irradiation for the controlled delivery of SB. SB/PVP solid dispersions were incorporated into the Gel/PVP nanogel matrix in the presence of NHS. FTIR and XRD confirmed the successful crosslinking and the amorphous nature of the formulation. TEM revealed pseudo-cubic nanoparticles below 50 nm. The system demonstrated pH-responsive release behavior, with 82% and 73% of SB released after 6 h at pH 4.5 and 5.5, respectively, markedly exceeding the release observed for free SB. Moreover, the SB/Gel/PVP nanogel exhibited enhanced antiproliferative activity against HepG2 cells compared with free SB (*P* < 0.05) and showed no evidence of acute hepatorenal toxicity in rats at an oral dose of 25 mg/kg, as confirmed by biochemical and histopathological assessments. However, its suitability for systemic or chronic therapeutic applications remains to be established.

## Supplementary Information

Below is the link to the electronic supplementary material.

Supplementary Material 1

## Data Availability

No datasets were generated or analysed during the current study.
