# Supplementary material for: Development of pH-responsive gelatin/PVP nanogel by gamma radiation for controlled delivery of silibinin
Source: Sci Rep. 2026 Jun 26;16:19614. doi: 10.1038/s41598-026-58355-8 (PMC13309560; doi:10.1038/s41598-026-58355-8)
Supplement: Supplementary file 1 — Supplementary Material 1 [file 41598_2026_58355_MOESM1_ESM.doc]

Table S1: P-values and confidence intervals for pairwise comparisons in the MTT assay.

Dependent Variable	Mean Difference (I-J)	Sig.	95% Confidence Interval	
			Lower Bound	Upper Bound	
Viability_7.8µg	LSD	Control	SB	5.75000*	0.001	3.1652	8.3348	
			SB/Gel/PVP	19.75000*	0.000	17.1652	22.3348	
		SB	Control	-5.75000*	0.001	-8.3348	-3.1652	
			SB/Gel/PVP	14.00000*	0.000	11.4152	16.5848	
		SB/Gel/PVP	Control	-19.75000*	0.000	-22.3348	-17.1652	
			SB	-14.00000*	0.000	-16.5848	-11.4152	
Viability_15.6µg	LSD	Control	SB	15.75000*	0.000	12.6986	18.8014	
			SB/Gel/PVP	45.00000*	0.000	41.9486	48.0514	
		SB	Control	-15.75000*	0.000	-18.8014	-12.6986	
			SB/Gel/PVP	29.25000*	0.000	26.1986	32.3014	
		SB/Gel/PVP	Control	-45.00000*	0.000	-48.0514	-41.9486	
			SB	-29.25000*	0.000	-32.3014	-26.1986	
Viability_31.25µg	LSD	Control	SB	25.00000*	0.000	21.7677	28.2323	
			SB/Gel/PVP	56.75000*	0.000	53.5177	59.9823	
		SB	Control	-25.00000*	0.000	-28.2323	-21.7677	
			SB/Gel/PVP	31.75000*	0.000	28.5177	34.9823	
		SB/Gel/PVP	Control	-56.75000*	0.000	-59.9823	-53.5177	
			SB	-31.75000*	0.000	-34.9823	-28.5177	
Viability_62.5µg	LSD	Control	SB	37.00000*	0.000	33.8008	40.1992	
			SB/Gel/PVP	67.00000*	0.000	63.8008	70.1992	
		SB	Control	-37.00000*	0.000	-40.1992	-33.8008	
			SB/Gel/PVP	30.00000*	0.000	26.8008	33.1992	
		SB/Gel/PVP	Control	-67.00000*	0.000	-70.1992	-63.8008	
			SB	-30.00000*	0.000	-33.1992	-26.8008	
Viability_125µg	LSD	Control	SB	84.00000*	0.000	81.4568	86.5432	
			SB/Gel/PVP	92.75000*	0.000	90.2068	95.2932	
		SB	Control	-84.00000*	0.000	-86.5432	-81.4568	
			SB/Gel/PVP	8.75000*	0.000	6.2068	11.2932	
		SB/Gel/PVP	Control	-92.75000*	0.000	-95.2932	-90.2068	
			SB	-8.75000*	0.000	-11.2932	-6.2068	
